# Diagnosis and Treatment of Pediatric Feeding Disorders: A Narrative Literature Review

**DOI:** 10.3390/children12030333

**Published:** 2025-03-06

**Authors:** Hugo Pergeline, Léo Gonnet, Arnaud Fernandez, Federico Solla, François Poinso, Jokthan Guivarch

**Affiliations:** 1 Medical School, Aix-Marseille University, 13385 Marseille, France; hugo.pergeline@ap-hm.fr (H.P.); francois.poinso@ap-hm.fr (F.P.); jokthan.guivarch@ap-hm.fr (J.G.); 2 Institut de Neurosciences de la Timone, UMR 7289, CNRS, Aix-Marseille University, 13005 Marseille, France; 3 Department of Child Psychiatry, Sainte Marguerite Hospital, 13008 Marseille, France; 4 Center for Diagnostic Evaluation of Child Development (CEDDE), 13008 Marseille, France; leo.gonnet@ens-lyon.fr; 5 Institut d’Histoire des Représentations et des Idées dans les Modernités, Ecole Normale Supérieure, UMR 5317, 69342 Lyon, France; 6 Department of Child and Adolescent Psychiatry, Children’s Hospitals Lenval, 57 Bd Californie, 06200 Nice, France; arnaud.fernandez@hpu.lenval.com; 7 Cognition Behaviour Technology (COBTEK) Lab, Université Côte d’Azur, 06100 Nice, France; 8 Pediatric Surgery Department, Pediatric Hospitals of Nice CHU-Lenval (HPNCL), 57 Bd Californie, 06200 Nice, France; 9 Healthcare Department, Link Campus University, 00165 Rome, Italy

**Keywords:** childhood eating disorders, oral disorders, feeding disorders, dysphagia, children, failure to thrive

## Abstract

**Background/Objectives:** The definitions of feeding disorders of infants and young children were historically based on a dichotomic organic/non-organic vision. Since 2019, a new definition of pediatric feeding disorders (PFDs) has reshaped the understanding of these disorders with a global vision. The aim of this study is to obtain a better understanding of the diagnostic criteria for general practice, both by exploring the evolution of classifications and by clearing the actual definition of PFDs and their possible treatments. **Methods:** We conducted a narrative review of the literature, including 36 articles about PFDs, excluding adolescents, anorexia nervosa, bulimia, pica, rumination, and specific neurodevelopmental or chronic pediatric disorders. We summarized these studies in three parts: the specific classifications for children before puberty, the current definition, and the clinical guidelines. **Results**: Concerning the history of the classifications, we summarized the studies of Chatoor and Kerzner and the older pediatric vision of failure to thrive. For the definition of pediatric feeding disorders, we presented this new category involving at least one out of four domains: medical, nutritional, feeding skills, or psychosocial. For the main clinical guidelines, we presented recommendations for both severe and common PFDs in each altered domain for use in daily practice. **Conclusions:** The new definition promotes a transdisciplinary vision of childhood feeding disorders, which considers each of the intricate domains of PFDs. Using common terminology for PFDs could help all healthcare providers, families, and researchers to better understand and address PFDs.

## 1. Introduction

Human feeding is a complex process of recent evolution [1], requiring the close interaction of the central and peripheral nervous systems, oropharyngeal functions, the cardiopulmonary system, the gastrointestinal tract, and all the craniofacial structures. Its good development begins in fetal life and evolves according to sensorimotor stimuli, especially through complementary feeding, a key stage of feeding evolution [2]. Normal learning of feeding is already a challenge for both infants, who are discovering their new feeding skills, and the caregiver, who is guiding them through their development [3]. This vital process can be altered by direct impairment of one of the necessary systems, one of the regulatory factors, or by a delay in neurodevelopmental development [4].

Feeding problems are one of the most common disorders faced by families: 25 to 45% of children will have at least one pediatric consultation for a feeding problem [5]. When asked, 50% of mothers report that one of their children does not eat enough, which represents 20 to 30% of children [6]. In certain populations, feeding difficulties are even more common, occurring in up to 80% of children with a neurodevelopmental disorder [7] and in 40 to 70% of children with other chronic illnesses [8]. In addition, over time, these early eating problems can lead to other feeding disorders in adolescents and adults, such as anorexia nervosa [9,10,11] and even obesity [12].

Historically, feeding disorders have been defined by each medical discipline independently of the others. The pediatric view was based on failure to thrive (FTT), specified as organic or inorganic [13], which can be difficult to distinguish in clinical practice [14,15]. The psychiatric view was based on the relationship between the infant and their caregiver during mealtime. In the late 1990s, Chatoor worked on feeding difficulties in infants and toddlers [16,17,18,19]. In 2002, they developed the Washington School Classification (WSC) based on the interactions between children and their caregivers [20,21,22]. All of these visions reflected a historical dichotomy between organic and non-organic subtypes, which is almost impossible to identify in clinical practice [23,24], even in the most recent world classifications, i.e., the DSM-5-TR [25] and the ICD-11 [26,27]. In the DSM-5-TR, the new diagnosis of Avoidant/Restrictive Food Intake Disorder (ARFID) excludes many children with feeding disorders, especially those with feeding skill impairments [28,29,30]. The ICD-11 uses the same diagnosis of ARFID, and the other codes that include other feeding disorders are in the category of symptoms, signs, or results of clinical examination, which is not sufficient to constitute a diagnosis in itself [26,31].

The first classification separating the organic/non-organic vision was established by Kerzner in 2009 [32,33]. In 2019, Goday et al. published a consensual definition of pediatric feeding disorder (PFD) that is easier to use and includes the different dimensions of feeding problems [4,34]. This new definition, which breaks from the historical organic/non-organic vision, allows for a better understanding between child psychiatrists and pediatricians.

Considering the recent changes and the new scientific interest in feeding disorders in children before puberty, we aimed to conduct a narrative review of the literature to help clinicians with the new definitions and to present some therapeutic guidelines. Our goal is to provide more efficient care through a better understanding and uniformity of diagnostic classifications.

The objectives of this narrative review are, first, to present the evolution of the classifications, second, to present the new definition of pediatric feeding disorders, and third, to present some of the clinical guidelines found in the literature to help clinicians in daily practice.

## 2. Materials and Methods

PFDs have only recently been studied, and due to the lack of clear definitions for years [24], there is a lack of consistency in the literature. Therefore, we decided to conduct a narrative review of the literature on feeding difficulties in infants and young children.

We searched Medline, PsychINFO, and PubMed for documents written in English or French from 1980 to 2022, as follows: randomized controlled trials, meta-analyses, case reports, and narrative reviews. Search parameters included the following combinations of key words: “feeding disorder”, “pediatric feeding disorder”, “picky eater” combined with the populations “toddlers”, “infants”, “children”. In addition, we reviewed the references of the identified articles and repeated our search on 13 February 2025 to ensure we had not missed any recent publication.

The exclusion criteria were studies that focused on eating disorders (anorexia nervosa and bulimia, which occur mostly in adolescents), as these are very different from the feeding disorders we see in infants and toddlers, and other specific feeding disorders (pica and rumination), as there are very specific feeding disorders that differ from the feeding difficulties we see in current practice, both in their presentation and in their care. We also excluded studies that focused mainly on populations with a specific chronic or neurodevelopmental disorder in order to help clinicians with a wide range of “common” feeding difficulties and not just the specificities of, for example, autism spectrum disorders. Moreover, we excluded studies that focused only on ARFID, as this specific condition does not match the more general presentation of pediatric feeding disorders and already has specific guidelines.

## 3. Results

We selected 140 full-text studies that met the inclusion and exclusion criteria. None of these studies included adolescent eating disorders (anorexia nervosa or bulimia nervosa).

From these studies, after reading, we excluded 104 studies that focused on very specific populations (cerebral palsy, autism spectrum disorder, patients with cardiovascular disease only, etc.) or very specific eating disorders for which guidelines already exist (ARFID, prepubertal anorexia nervosa).

Finally, we selected 36 main studies for this narrative review of the literature and summarized them into these three chapters as follows:

- The different feeding disorders of infants and toddlers described since 1980;
- The current definition of pediatric feeding disorders;
- The clearest clinical guidelines.

The list of articles used for the narrative review of the literature is included in Appendix A (see below).

### 3.1. The Special Classifications for Children

Chatoor et al. first described patients with feeding refusal and failure to thrive (FTT), described as separation disorder [17], then used the definition of infantile anorexia nervosa [18,35,36]. Chatoor already pointed out the lack of consensus regarding the feeding difficulties of children and the terms used [21]. His work led to a classification of feeding disorders in infants and young children with a rating scale, the Chatoor Feeding Scale [16]. The WSC [22] defined six distinct disorders:

- A feeding disorder of state regulation;
- A feeding disorder of caregiver–infant reciprocity [18];
- Infantile anorexia [36];
- Posttraumatic feeding disorder [19];
- Sensory food aversion;
- A feeding disorder associated with a concurrent medical condition.

The WSC was the first to describe specific feeding disorders in infants and toddlers [20] and has been used in the Diagnostic Classification of Mental and Developmental Disorders of Infancy and Early Childhood (DC: 0–3) [37]. However, Chatoor’s classification focused specifically on the caregiver–child relationship without an integrative view that could express the interdisciplinary nature of feeding disorders.

In its latest version, the DC: 0–5, the classification of feeding disorders is based on the new approach by Kerzner et al. (2015) as on the initial complaints of the parents [38]. For the first time, Kerzner tried to break away from the organic/non-organic vision. They aimed to create a classification for the primary care provider that was easy to use, integrated organic and behavioral views, included the severity spectrum, and incorporated parents’ feeding styles [38]. Therefore, they defined four feeding styles with different influences on the child’s feeding behavior [12] (Table 1). Thus, Kerzner first described red flags that are both organic and behavioral (Table 1). They then defined three categories of feeding problems: restricted appetite, selective appetite [39], or fear of feeding, each with subcategories. Misperception can be involved in all three categories and can improve with parental guidance (see clinical guidelines, Section 3.3). There is also the possibility of a link to an organic disorder, including delayed mental development or motor disorders in cases of selective appetite and pain in cases of fear of eating. Other subcategories are close to those described by the WSC.

To sum up, this definition was the first to focus on the clinical presentation of the patient and include suggestions for care. However, the authors outlined the limitations of their classification, particularly the lack of continuum between definitions and subcategories, which could complicate both care and diagnosis by multiplying the diagnosed disorders [33].

### 3.2. The Definition of Pediatric Feeding Disorders

In 2019, Goday et al. published an updated definition of pediatric feeding disorders (PFDs): “Impaired oral intake that is not age-appropriate, and is associated with medical, nutritional, feeding skill, and/or psychosocial dysfunction” [4]. They classified acute and chronic PFDs as the duration being less or more than 3 months. The authors proposed diagnostic criteria for PFDs (Table 2).

The most important component of this definition is the introduction of four different domains that are intimately related, since the impairment of one could cause the dysfunction of the others, making it impossible to know which was impaired first. The authors emphasize the importance of always assessing the four domains in order not to underestimate any of the dysfunctions and to address the symptoms in their totality, with complementary domain-specific treatments for optimal care. However, whatever the domain affected, the generic PFD diagnosis is always retained.

With regard to medical factors, the authors specify the frequent combination of functional and/or structural impairments that could cause a PFD. For instance, airway disorders could lead to dysregulation of suck–swallow–breath coordination and alter feeding ability. Moreover, gastrointestinal diseases could also have a direct impact on feeding ability [40]. Additionally, children with neurological or neurodevelopmental disorders may have nutritional needs that exceed their motor skills to feed themselves [7,41].

In terms of nutritional factors, the selective or restrictive diets of some children with PFDs pose a risk of malnutrition or micronutrient deficiencies, especially in children with selective appetites, as described by Kerzner [38]. Nutritional impairment may also lead to obesity, especially if a child has a selective appetite for palatable foods.

Regarding the feeding skill factors, Goday classified them according to the oral or pharyngeal location of the impairment and whether the impairment is motor, structural, or sensory. Specifically, in neurodevelopmental disorders, developmental delay can lead to a PFD by directly impairing oral motor skills. In addition, sensorimotor aversive experiments during feeding development or limited/insufficient feeding experiments could alter feeding skills. Oral sensory processing disorder may manifest as a PFD with altered feeding skills [29]. Pharyngeal impairments may alter swallowing. The authors describe three skill-based dysfunctions: unsafe oral feeding, delayed feeding skills, and inefficient oral feeding [4].

Moreover, aversive effects of psychosocial factors may lead to a PFD or contribute to its maintenance, even if another domain has been corrected. Goday identifies four impairments: developmental factors, mental and behavioral health problems, social factors, and environmental factors [4]. These impairments can result in one or more psychosocial dysfunctions:

- Learned feeding aversions with avoidance behaviors;
- Child and/or caregiver stress and distress;
- Disruptive behaviors;
- Over-selection of food;
- Grazing with a false sense of satiety;
- Caregiver use of inappropriate strategies [42].

### 3.3. Clinical Guidelines

As PFDs were only properly defined in 2019, the guidelines found in the literature are not uniform. We describe here the most relevant ones, selected first for their usefulness in severe PFDs and second for their ease of use by clinicians.

A more recent review, based on Kerzner’s studies, proposed an algorithm to firstly check the nutritional status and both organic and behavioral red flags in order to distinguish severe from common PFDs [43].

#### 3.3.1. For Severe PFDs

Sharp et al. conducted a meta-analysis in 2017 to evaluate intensive multidisciplinary interventions for severe PFDs [44]. In the context of intensive inpatient programs for children requiring non-oral enteral nutrition, one of the success criteria was the removal of enteral nutrition. One of the therapeutic methods was tube weaning, i.e., progressive reduction in enteral nutrition below the recommended daily allowance to induce hunger. They provided recommendations for standard intensive care for severe PFDs:

- Multidisciplinary intervention should include psychotherapy, medical, nutritional, speech, and occupational therapy;
- Behavioral intervention is one of the core treatments to increase oral intake but also to avoid negative reinforcements;
- Families must be involved in care to maintain changes at home;
- Care discharge lays on a slow transition to home with a long-term follow-up.

Regarding behavioral therapies, Sharp specifies them through escape–extinction techniques and positive reinforcements that involve caregivers to maintain at home [45]. Oro-motor exercises and oral sensory desensitization may also play a role in improving oral strength and motor control of the orofacial muscles.

However, this meta-analysis included few randomized studies compared to non-randomized studies. In addition, the recommended treatments lack uniformity and standardization in terms of their effectiveness. Moreover, it only concerns intensive hospital care for severe PFD patients with enteral nutrition, who are not the patients most commonly encountered in clinical practice.

#### 3.3.2. For Common PFDs in Daily Practice

○ Regarding psychosocial factors, Kerzner provided recommendations for care according to the “old” classification [38]. Although the classification is no longer accurate, the guidelines can still guide behavioral care, stimulating appetite and inducing environmental changes with positive reinforcement [46]. All of these recommendations are based on Kerzner’s classification on clinical presentations (misperception, limited appetite, selective intake, and fear of feeding) [39], which are still useful to lead behavioral treatments [47].
  ○ Regarding misperception, which can be associated with each presentation, the treatment is first based on the restoration of the hunger–satiety cycle through a simple behavioral method: a good feeding rhythm (i.e., five meals a day, including snacks for infants) and frequent presentation of the food (8 to 15 times) [48].
  ○ For limited appetite, enriching the food can help.
  ○ For selective appetite, chaining (progressive presentation of different foods with similar characteristics) can be used [48,49].
  ○ For feeding anxiety, transition to a spoon may help, and the authors emphasize the possible use of anxiolytics or cognitive and behavioral psychotherapy to treat excessive worry [50,51].

On the other hand, forcing a child to eat is always described as an inefficient strategy [38,42].

In recent studies [52,53], escape–extinction techniques, which can be commonly used for these psychosocial factors, seem to be more efficient when combined with reinforcement of alternative behavior and other noncontingent reinforcement, i.e., the delivery of reinforcement independent of behavior.

○ Regarding eating skills, good feeding development requires early exposure to sensorimotor stimuli, especially during complementary feeding.
  ○ In premature infants, early oral and peri-oral stimulation with postural support and sensory stimulation are recommended to reduce the duration of non-oral enteral feeding and prevent feeding delays [54].
  ○ Sensory processing disorders can also be assessed with a sensory profile and treated specifically with the help of speech–language therapists or psychomotricians [55].
○ Concerning nutritional factors, the needs of infants and young children differ from those of adults. After six months, breast milk is unable to provide enough iron to meet the infant’s nutritional needs [2,3]. The main goal is to prevent malnutrition, which is an independent factor of morbidity and mortality.
  ○ If malnutrition is already present, refeeding strategies are needed, favoring oral intake. It is possible to enrich the milk or the food or to use oral nutritional supplements [2,16,31]. Dietitians are key experts in this domain.
  ○ As a second intention, non-oral enteral feeding can be used, including nasogastric tubes, gastrostomy [56], and jejunostomy. Weaning is a clear challenge in PFDs because enteral nutrition has traumatic, infectious, metabolic, and behavioral side effects. It is also important to prevent refeeding syndrome [57].
  ○ With regard to nutritional factors, we can also mention a current study on possible orexigenic stimulations, especially with the ghrelin receptor [58], but this work is still in progress.
○ Finally, regarding medical factors, the physician can make the diagnosis of a PFD and identify the altered domains according to the recent definition. They also need to identify the red flags and address them. Physicians from different specialties could work together for optimal care according to their expertise, such as primary care pediatricians, pediatric gastroenterologists, otolaryngologists, pediatric pneumologists, pediatric neurologists, and child psychiatrists [4,44].
○ Moreover, it is important to take care of the whole family system [4,38], including the training of the caregivers in the behavioral techniques used, so that they can be continued at home [4,59], as well as to take care of their own anxiety to prevent environmental aversive factors [38,42,59]. A link with the child’s school can also improve the social integration of children with PFDs [4].

## 4. Discussion and Conclusions

In this narrative review, we organized the concept of pediatric feeding disorders into three chapters to help clinicians understand the historical evolution of feeding disorder assessment, the current definition of PFDs, and the most important guidelines for clinical practice.

With a wide variety of definitions, goals, and populations (many articles including pre- and post-pubertal children), we chose to conduct a narrative review rather than a systematic review due to obvious bias from too many different study types.

After years of undefined terms and a historical organic/nonorganic dichotomy, PFDs now have clearly defined diagnostic criteria. They involve the systematic examination of four interrelated domains: medical, nutritional, feeding skills, and psychosocial. This new terminology promotes a transdisciplinary vision of childhood feeding disorders that allows us to consider each of the intricate domains of PFDs. With this common definition, which may be adopted worldwide, we can hope for an effective impact on clinical, educational, and research areas.

Using a shared terminology for PFDs could help all healthcare providers, from psychiatrists to gastroenterologists, facing a wide variety of clinical situations, as well as ensure that patients receive the best care. Thus, if the four domains are systematically assessed, no single factor that may lead to a PFD is forgotten, and we can implement domain-specific treatment. Therapeutic efficacy still needs more studies for a better evaluation, and the guidelines presented here lack uniformity and evidence; however, according to the impaired domains, individualized care is recommended for children suffering from PFDs [4,38,49,50]. We need to improve these guidelines and their level of scientific evidence.

The definition of PFDs also allows families to better understand their child’s feeding difficulties, both through a more complete assessment of their child’s feeding skills and through appropriation of their own caregiver’s feeding style [32]. Moreover, it allows parents to access family training and psychoeducation by professionals who are more aware of PFDs. For families, the four-domain systematic assessment avoids a stigmatizing judgment of their caregiving, as the psychosocial domain is the most obvious in consultation but is rarely the only one [4].

Finally, the use of a shared terminology can promote research on these treatments, since a common definition of the disorder would lead to less heterogeneity in the results, as suggested in 2022 by Sharp et al. [60]. They propose a case report form to systematically evaluate both the diagnosis and treatment and to standardize care worldwide with a simple and useful clinical tool. This would also allow for more consistent and transdisciplinary research, as PFD is a trans-diagnostic term that encompasses both physiological and functional impairments.

Although healthcare providers have easy access to normal feeding recommendations, PFDs and their interdisciplinary care are still under-recognized. With this paper, we hope to clarify clinical management criteria and promote research to develop specific guidelines.

## Figures and Tables

**Table 1:** Classification of pediatric feeding disorders according to Kerzner et al. (2015) [38].

A. Parental feeding styles:
1. Responsive; 2. Controlling; 3. Indulgent; 4. Neglectful.
B. Red flags:
1. Dysphagia; 2. Aspiration; 3. Apparent pain at feeding; 4. Developmental delay; 5. Growth failure; 6. Food fixation; 7. Harmful feeding; 8. Anticipatory gagging.
C. Categories of feeding problems (misperception can be involved in all three categories):
1. Restricted appetite; 2. Selective appetite; 3. Fear of feeding.

**Table 2:** Diagnostic criteria of pediatric feeding disorders according to Goday et al. (2019) [4].

A. A disturbance in oral intake of nutrients, inappropriate for age, lasting ≥ 2 weeks, and associated with one or more of the following:
a. Medical dysfunction, as evidenced by any of the following:
i. Cardiorespiratory compromise during oral feeding; ii. Aspiration or recurrent aspiration pneumonitis.
b. Nutritional dysfunction, as evidenced by any of the following:
i. Malnutrition; ii. Specific nutrient deficiency or significantly restricted intake of one or more nutrients resulting from decreased dietary diversity; iii. Reliance on enteral feeds or oral supplements to sustain nutrition and/or hydration.
c. Feeding skill dysfunction, as evidenced by any of the following:
i. Need for texture modification of liquid or food; ii. Use of modified feeding position or equipment; iii. Use of modified feeding strategies.
d. Psychosocial dysfunction, as evidenced by any of the following:
i. Active or passive avoidance behaviors by child when feeding or being fed; ii. Inappropriate caregiver management of child’s feeding and/or nutrition needs; iii. Disruption of social functioning within a feeding context; iv. Disruption of caregiver–child relationship associated with feeding.
B. Absence of the cognitive processes consistent with eating disorders and pattern of oral intake is not due to a lack of food or congruent with cultural norms.

## Data Availability

The original contributions presented in this study are included in the article/Appendix A. Further inquiries can be directed to the corresponding author.

## Abbreviations

The following abbreviations are used in this manuscript:

ARFID	Avoidant/Restrictive Food Intake Disorder
DSM-5-TR	Diagnostic and Statistical Manual of Mental Disorders, 5th Edition, Text Revision
FTT	Failure to thrive
ICD-11	International Classification of Diseases 11th Revision
PFD	Pediatric feeding disorder
WSC	Washington School Classification

## Appendix A

**Table A1:** Table of articles used for the narrative review in chronological order of publication.

Year of Publication and Citation Reference	Title	Type of Study
1988 [10]	Bryant-Waugh R., Knibbs J., Fosson A., Kaminski Z., & Lask B. (1988). Long term follow up of patients with early onset anorexia nervosa. *Archives of Disease in Childhood*, 63(1), 5–9.	Follow-up study for an average of 7 years of 44 children aged 7 to 13 years diagnosed with early-onset anorexia nervosa.
1989 [15]	Wilcox W. D., Nieburg P., & Miller D. S. (1989). Failure to thrive: A continuing problem of definition. *Clinical pediatrics*, *28*(9), 391–394.	Narrative review of the literature on FTT definitions.
1997 [16]	Chatoor, I. Getson P., Menvielle E., Brasseaux C., O’Donnell R., Rivera Y., & Mrazek D. A. (1997). A feeding scale for research and clinical practice to assess mother—Infant interactions in the first three years of life. *Infant Mental Health Journal*, *18*(1), 76–91.	Validation study of Chatoor’s feeding scale.
1998 [17]	Chatoor I., Ganiban J., Colin V., Plummer N., & Harmon R. J. (1998). Attachment and Feeding Problems: A Reexamination of Nonorganic Failure to Thrive and Attachment Insecurity. *Journal of the American Academy of Child & Adolescent Psychiatry*, *37*(11), 1217–1224.	Observational study of attachment insecurity in picky eaters, infantile anorexia, and healthy eaters in 101 toddlers (12 to 37 months).
1998 [36]	Chatoor I., Hirsch R., Ganiban J., Persinger M., & Hamburger E. (1998). Diagnosing Infantile Anorexia: The Observation of Mother-Infant Interactions. *Journal of the American Academy of Child & Adolescent Psychiatry*, *37*(9), 959–967.	Observational study of mother–child interactions among picky eaters, infantile anorexics, and healthy eaters in 34 toddlers (12 to 37 months).
2000 [18]	Chatoor I., Ganiban J., Hirsch R., Borman-Spurrell E., & Mrazek D. A. (2000). Maternal Characteristics and Toddler Temperament in Infantile Anorexia. *Journal of the American Academy of Child & Adolescent Psychiatry*, *39*(6), 743–751.	Observational study of mother–infant attachment in picky eaters, infantile anorexia, and healthy eaters in 102 toddlers.
2001 [19]	Chatoor I., Ganiban J., Harrison J., & Hirsch R. (2001). Observation of Feeding in the Diagnosis of Posttraumatic Feeding Disorder of Infancy. *Journal of the American Academy of Child & Adolescent Psychiatry*, *40*(5), 595–602.	Observational study of the feeding resistance scale in posttraumatic feeding disorder, infantile anorexia, and healthy eaters in 90 infants < 32 months.
2001 [11]	Kotler L. A., Cohen P., Davies M., Pine D. S., & Walsh B. T. (2001). Longitudinal Relationships Between Childhood, Adolescent, and Adult Eating Disorders. *Journal of the American Academy of Child & Adolescent Psychiatry*, *40*(12), 1434–1440.	Longitudinal observational study of 800 children who developed adolescent eating disorders (anorexia and bulimia nervosa), with initial assessment in childhood < 10 years.
2002 [21]	Chatoor I. (2002). Feeding disorders in infants and toddlers: Diagnosis and treatment. *Child and Adolescent Psychiatric Clinics*, *11*(2), 163–183.	Narrative review of the literature for a new definition of feeding disorders in infants and toddlers.
2004 [6]	Carruth B. R., Ziegler P. J., Gordon A., & Barr S. I. (2004). Prevalence of picky eaters among infants and toddlers and their caregivers’ decisions about offering a new food. *Journal of the American Dietetic Association*, *104*, 57–64.	Observational study of the prevalence of picky eaters among 3022 infants and toddlers < 24 months.
2006 [24]	Olsen E. M. (2006) Failure to Thrive: Still a Problem of Definition. *Clin Pediatr (Phila)*, 45 (1): 1–6	Narrative review of the literature on FTT definitions.
2006 [49]	Fishbein M., Cox S., Swenny C., Mogren C., Walbert L., & Fraker C. (2006). Food chaining: A systematic approach for the treatment of children with feeding aversion. *Nutrition in clinical practice*, *21*(2), 182–184.	Evaluation of food chaining in ten children < 14 years.
2006 [42]	Galloway A. T., Fiorito L. M., Francis L. A., & Birch L. L. (2006). ‘Finish your soup’: Counterproductive effects of pressuring children to eat on intake and affect. *Appetite*, *46*(3), 318–323.	Evaluation of pressure versus no-pressure feeding conditions in 27 children (age 3 to 5 years).
2008 [39]	Jacobi C., Schmitz G., & Agras W. S. (2008). Is picky eating an eating disorder? *International Journal of Eating Disorders*, *41*(7), 626–634.	Observational study of children’s eating behavior in 426 children (age 7–12 years).
2010 [35]	Ammaniti M., Lucarelli L., Cimino S., D’Olimpio F., & Chatoor I. (2010). Maternal psychopathology and child risk factors in infantile anorexia. *International Journal of Eating Disorders*, *43*(3), 233–240.	Observational study of the association between maternal psychological symptoms and infantile anorexia in 371 pairs (children < 36 months and their mothers) in infantile anorexia vs healthy controls.
2010 [45]	Sharp W. G., Jaquess D. L., Morton J. F., & Herzinger C. V. (2010). Pediatric Feeding Disorders: A Quantitative Synthesis of Treatment Outcomes. *Clinical Child and Family Psychology Review*, *13*(4), 348–365.	Systematic review of the literature on the treatment of pediatric feeding disorders.
2012 [52]	Addison L.R., Piazza C.C., Patel M.R., Bachmeyer M. H., Rivas K. M., Milnes S.M., Oddo J. (2012). A comparison of sensory integrative and behavioral therapies as treatment for pediatric feeding disorders. *Journal of Applied Behavior Analysis, 45 (3):* 455–471	Comparative study of escape–extinction plus noncontingent reinforcement with sensory integration therapy as treatment for feeding problems in two children.
2012 [9]	Ammaniti M., Lucarelli L., Cimino S., D’Olimpio F., & Chatoor I. (2012). Feeding disorders of infancy: A longitudinal study to middle childhood. *International Journal of Eating Disorders*, *45*(2), 272–280.	Longitudinal study of 142 children (age 2 years at assessment) diagnosed with feeding disorders and their mothers, followed for a mean of 5 years.
2012 [29]	Farrow C. V., & Coulthard H. (2012). Relationships between sensory sensitivity, anxiety and selective eating in children. *Appetite*, *58*(3), 842–846.	Observational study of 95 children (ages 5 to 10 years) examining the relationship between reported selective eating behaviors, child anxiety, and child sensory sensitivity.
2012 [12]	Hennessy E., Hughes S. O., Goldberg J. P., Hyatt R. R., & Economos C. D. (2012). Permissive Parental Feeding Behavior Is Associated with an Increase in Intake of Low-Nutrient-Dense Foods among American Children Living in Rural Communities. *Journal of the Academy of Nutrition and Dietetics*, *112*(1), 142–148.	Observational study of 99 children (ages 6–11 years) examining the relationship between permissive feeding style and the intake of nutrient-poor foods.
2012 [26]	Uher R., & Rutter M. (2012). Classification of feeding and eating disorders: Review of evidence and proposals for ICD-11. *World Psychiatry*, *11*(2), 80–92.	Narrative review of the literature summarizing the changes in the classification of feeding and eating disorders.
2013 [13]	Yoo S. D., Hwang E.-H., Lee Y. J., & Park J. H. (2013). Clinical Characteristics of Failure to Thrive in Infant and Toddler: Organic vs. Nonorganic. *Pediatric Gastroenterology, Hepatology & Nutrition*, *16*(4), 261–268.	Retrospective study of 123 infants and toddlers under 24 months of age diagnosed with FTT to compare clinical differences and causes.
2014 [20]	Cascales T., Olives, J.-P. Bergeron, M., Chatagner A., & Raynaud J.-P. (2014). Les troubles du comportement alimentaire du nourrisson: Classification, sémiologie et diagnostic. *Annales Médico-psychologiques, revue psychiatrique*, *172*(9), 700–707.	Narrative review of the literature focusing on the Washington classification.
2014 [30]	Nicely T. A., Lane-Loney S., Masciulli E., Hollenbeak C. S., & Ornstein R. M. (2014). Prevalence and characteristics of avoidant/restrictive food intake disorder in a cohort of young patients in day treatment for eating disorders. *Journal of Eating Disorders*, *2*(1), 21.	Retrospective study on 173 patients hospitalized for eating disorders to determine prevalence and clinical characteristics of ARFID.
2014 [51]	Wilken M., & Bartmann P. (2014). Posttraumatic Feeding Disorder in Low Birth Weight Young Children: A Nested Case–Control Study of a Home-Based Intervention Program. *Journal of Pediatric Nursing*, *29*(5), 466–473.	Evaluation of a home-based feeding disorder intervention program in 21 children with posttraumatic feeding disorders.
2015 [38]	Kerzner B., Milano K., MacLean W. C. Jr, Berall G., Stuart S., & Chatoor I. (2015). A Practical Approach to Classifying and Managing Feeding Difficulties. *Pediatrics*, *135*(2), 344–353.	Narrative review of the literature defining a new classification of feeding disorders.
2017 [33]	Estrem, H. H., Pados, B. F., Park, J., Knafl, K. A., & Thoyre, S. M. (2017). Feeding problems in infancy and early childhood: Evolutionary concept analysis. *Journal of Advanced Nursing*, *73*(1), 56–70.	Narrative review of the literature on the conceptualization of feeding problems.
2017 [44]	Sharp W. G., Volkert V. M., Scahill L., McCracken C. E., & McElhanon B. (2017). A Systematic Review and Meta-Analysis of Intensive Multidisciplinary Intervention for Pediatric Feeding Disorders: How Standard Is the Standard of Care? *The Journal of Pediatrics*, *181*, 116–124.	Systematic review and meta-analysis of the treatment of children with chronic refusal to eat.
2018 [46]	Chatoor I., Hommel S., Sechi C., & Lucarelli L. (2018). Development of the Parent-Child Play Scale for Use in Children with Feeding Disorders. *Infant Mental Health Journal*, *39*(2), 153–169.	Development of a parent–child play scale to complement the feeding scale.
2019 [53]	Berth D.P., Bachmeyer M. H., Kirkwood C.A., Mauzy 4th C.R., Retzlaff B. J., Gibson A.L. (2019). Noncontingent and differential reinforcement in the treatment of pediatric feeding problems. *Journal of Applied Behavior Analysis, 52 (3)*, 622–641.	Case series of five children reporting the effect of non-contingent and differential reinforcement in the treatment of pediatric eating disorders.
2019 [4]	Goday P. S., Huh S. Y., Silverman A., Lukens C. T., Dodrill P., Cohen S. S., Delaney A. L., Feuling M. B., Noel R. J., Gisel E., Kenzer A., Kessler D. B., Kraus de Camargo O., Browne J., & Phalen J. A. (2019). Pediatric Feeding Disorder: Consensus Definition and Conceptual Framework. *Journal of Pediatric Gastroenterology and Nutrition*, *68*(1), 124–129.	Narrative review of the literature with a new consensus definition of pediatric feeding disorders.
2019 [47]	Milano K., Chatoor I., & Kerzner B. (2019). A Functional Approach to Feeding Difficulties in Children. *Current Gastroenterology Reports*, *21*(10), 51.	Narrative review of the management of feeding difficulties according to Kerzner’s classification.
2020 [48]	Zeleny J. R., Volkert V. M., Ibañez V. F., Crowley J. G., Kirkwood C. A., & Piazza C. C. (2020). Food preferences before and during treatment for a pediatric feeding disorder. *Journal of Applied Behavior Analysis*, *53*(2), 875–888.	Evaluation of food preferences in three children admitted for feeding disorder before and after repeated exposure to food.
2021 [34]	Kovacic K., Rein L. E., Szabo A., Kommareddy S., Bhagavatula P., & Goday P. S. (2021). Pediatric Feeding Disorder: A Nationwide Prevalence Study. *The Journal of Pediatrics*, *228*, 126–131.	Retrospective cohort study of medical databases from Arizona and Wisconsin, identifying an approximate prevalence of 3%.
2022 [60]	Sharp W. G., Silverman A., Arvedson J. C., Bandstra N. F., Clawson E., Berry R. C., McElhanon B. O., Kozlowski A. M., Katz M., Volkert V. M., Goday P. S., & Lukens C. T. (2022a). Toward Better Understanding of Pediatric Feeding Disorder: A Proposed Framework for Patient Characterization. *Journal of Pediatric Gastroenterology and Nutrition*, *75*(3), 351–355.	Narrative review of the literature proposing a new approach to standardize the assessment and diagnosis of pediatric feeding disorders using a case report form.
2024 [43]	Saure C., Zonis L. N., Gonzalez Sanguinetti X., Kovalskys I. (2024). Feeding difficulties in childhood: A narrative review. *Archivos Argentinos de Pediatria, 122 (5).*	Narrative review of the literature on childhood feeding difficulties.
